# Electroacupuncture at Baliao points added to anorectal biofeedback for spinal cord injury–related neurogenic bowel dysfunction: a single-center prospective preference-allocated comparative study

**DOI:** 10.3389/fmed.2026.1866886

**Published:** 2026-08-12

**Authors:** Mingxuan Liao, Xiao Huang, Dan Mo

**Affiliations:** General Hospital of The Yangtze River Shipping, Wuhan Brain Hospital, Wuhan, China

**Keywords:** anorectal biofeedback, Baliao points, electroacupuncture, heart rate variability, neurogenic bowel dysfunction, rehabilitation, spinal cord injury

## Abstract

**Background:**

Neurogenic bowel dysfunction (NBD) after spinal cord injury (SCI) remains difficult to manage in rehabilitation practice. Anorectal biofeedback targets defecatory coordination, whereas electroacupuncture at Baliao points may provide an additional sacral-region intervention. This study evaluated short-term between-group associations when Baliao electroacupuncture was added to anorectal biofeedback; it was not designed to isolate an electroacupuncture-specific effect.

**Methods:**

This single-center prospective preference-allocated comparative study enrolled 106 consecutive patients with SCI-related NBD between March 2024 and August 2025. Fifty-two received anorectal biofeedback alone and 54 received Baliao electroacupuncture plus biofeedback. The protocol scheduled 18 treatment sessions over approximately 6 weeks, with assessments at baseline, week 6, and week 12. Week 6 NBD score was the sole primary comparison. Covariate-adjusted models, propensity-score overlap weighting, mixed-effects models, and inverse-probability-of-follow-up weighting were supplemented by *post-hoc* model-stability, multiplicity, severity-transition, balloon-expulsion, and injury-completeness sensitivity analyses.

**Results:**

Week 6 assessments were completed by 51/52 patients in the biofeedback group and 52/54 in the combined-treatment group. NBD score decreased more in the combined-treatment group (change, −5.2 ± 2.9 vs. −2.9 ± 1.9 points; adjusted difference, −2.1; 95% CI, −3.2 to −1.1; *p* < 0.001). In a *post-hoc* Holm sensitivity analysis at week 6, PAC-QOL, anal relaxation during straining, and RMSSD remained below 0.05 across non-primary outcomes, whereas the small spontaneous-bowel-movement difference did not (Holm-adjusted *p* = 0.085). The primary NBD estimate was similar in baseline-adjusted, parsimonious, full, overlap-weighted, and 2,000-replicate bootstrap analyses. At week 12, only the NBD-score difference remained below 0.05 after Holm adjustment.

**Conclusion:**

In this preference-allocated, non-randomized study, adding Baliao electroacupuncture to biofeedback was associated with a modestly larger short-term reduction in NBD score. Secondary and physiological findings were exploratory, and the design does not establish an electroacupuncture-specific contribution, causal superiority, or durable benefit. Confirmation requires prospectively registered, adequately powered randomized trials with attention-matched controls, blinded outcome assessment, and longer follow-up.

## Introduction

Neurogenic bowel dysfunction is a persistent and often under-addressed consequence of spinal cord injury (SCI). It usually presents as constipation, prolonged evacuation, incomplete emptying, fecal incontinence, or dependence on assisted bowel care; these symptoms arise from disrupted autonomic and somatic control of colonic, rectal, and pelvic floor function (1–3). Although bowel dysfunction is not always the most visible impairment after SCI, patients frequently rank bowel care as one of the most burdensome aspects of daily living (4, 5).

The clinical burden of SCI-related NBD extends beyond stool frequency. Bowel programs can be time-consuming, socially restrictive, and difficult to sustain, particularly when patients require digital stimulation, rectal medication, or manual assistance (6, 7). Cross-sectional and longitudinal data suggest that bowel symptoms are closely related to health-related quality of life, and that constipation-related symptoms may worsen with time since injury (8–10). In a Chinese SCI cohort, bowel dysfunction was associated with poorer quality of life and greater daily-management burden (11).

Contemporary guidelines emphasize individualized bowel management, structured assessment, and stepwise escalation of treatment according to symptom pattern, neurologic level, patient preference, and response to conservative care (7, 12). Common approaches include dietary and fluid optimization, scheduled bowel programs, oral or rectal medications, abdominal massage, digital rectal stimulation, transanal irrigation, and, in selected cases, surgical options (3, 5, 6). However, many interventions remain supported by limited or heterogeneous evidence, and the field continues to need pragmatic rehabilitation studies that combine patient-reported outcomes with objective functional assessment (13).

The NBD score is a widely used symptom-based instrument developed for clinical assessment of colorectal and anal dysfunction in SCI. It has demonstrated reliability, construct validity, and sensitivity to change, although internal consistency was limited in one validation study and a definitive minimal clinically important difference has not been established (14, 15). Because bowel symptoms substantially affect daily functioning and well-being, quality-of-life instruments are also important. The Patient Assessment of Constipation Quality of Life questionnaire (PAC-QOL) was developed to measure the everyday burden of constipation and has been widely applied in constipation-related research (16).

Anorectal biofeedback is a rehabilitation technique that uses visual or auditory feedback to retrain pelvic floor and anorectal coordination. In non-neurogenic dyssynergic defecation, biofeedback is generally considered an evidence-based treatment because it addresses the abnormal rectoanal coordination underlying impaired evacuation (17, 18). Evidence in SCI-related NBD is smaller and more selective, but a case–control study in incomplete motor SCI reported improvements in bowel symptoms, quality of life, and anorectal physiological measures after biofeedback (19). Earlier work in neurogenic fecal incontinence was less encouraging, suggesting that lesion completeness, residual voluntary control, and the dominant bowel phenotype may influence response (20).

Electroacupuncture has been explored as a neuromodulatory adjunct in SCI rehabilitation. Systematic reviews have suggested possible benefits for selected SCI-related outcomes, although methodological limitations and heterogeneity remain substantial (21). A small prospective observational study reported changes in bladder and bowel function after electroacupuncture in traumatic SCI (22). Baliao points (BL31-BL34) are located over the sacral region and are commonly selected in Chinese rehabilitation practice for pelvic and anorectal disorders. A randomized study in a non-SCI anorectal condition and a multicenter trial protocol motivate further evaluation of Baliao-region electroacupuncture but do not establish efficacy in SCI-related NBD (23, 24).

The physiological rationale for combining Baliao electroacupuncture with anorectal biofeedback is that the two interventions may target different but complementary components of bowel control. Biofeedback directly trains voluntary anorectal coordination, whereas sacral-region electroacupuncture may modulate afferent and autonomic inputs relevant to pelvic organ function (18, 25). Heart rate variability (HRV), particularly RMSSD, provides a non-invasive exploratory index of parasympathetic-related autonomic modulation, although it cannot by itself establish a causal neural pathway (26–28).

Previous studies have separately examined anorectal biofeedback in selected patients with SCI, electroacupuncture in small SCI cohorts, and Baliao-region stimulation in non-SCI anorectal conditions. The present study did not isolate an electroacupuncture-specific effect. Its contribution was a pragmatic, hypothesis-generating comparison of two preference-allocated rehabilitation pathways using clinical, anorectal, and short-term follow-up outcomes. We therefore evaluated whether the combined-treatment pathway was associated with different short-term outcomes from biofeedback alone, without assuming a causal additive effect.

## Materials and methods

### Study design and setting

This was a single-center prospective preference-allocated comparative rehabilitation study conducted in the Rehabilitation Medicine Department, General Hospital of The Yangtze River Shipping, Wuhan Brain Hospital, Wuhan, China. Consecutive eligible patients were enrolled from March 11, 2024, through August 28, 2025. No study-generated random sequence was used. After eligibility confirmation and baseline assessment, all participants received standardized information regarding the procedures, potential benefits, and possible adverse effects of biofeedback alone and electroacupuncture plus biofeedback. Participants who accepted electroacupuncture entered the combined-treatment group, whereas those who preferred not to undergo electroacupuncture received biofeedback alone. Allocation was finalized before the first intervention session, and switching was not routinely permitted. Treatment choice was documented clinically; however, the specific factors influencing treatment selection were not prospectively collected in a standardized manner. Consequently, the relative contributions of treatment preference, expectations, perceived burden, cost, and logistical considerations could not be quantified.

Reporting was guided by the TREND statement for nonrandomized intervention studies and the STRICTA recommendations for acupuncture interventions (29, 30). The protocol was reviewed and approved by the Ethics Committee of General Hospital of The Yangtze River Shipping/Wuhan Brain Hospital [approval number: ytz260233]. Written informed consent was obtained from all participants before enrollment. The study was not prospectively registered. At study initiation, registry entry was not mandated under the applicable local/institutional process for this non-randomized comparative rehabilitation study; nevertheless, the absence of prospective registration is reported transparently and acknowledged as a limitation.

No formal *a priori* sample-size calculation was performed. The sample comprised all consecutive eligible participants enrolled during the study period; precision is therefore presented using 95% confidence intervals rather than retrospective observed power.

### Participants

Patients were screened from the rehabilitation department among adults with a documented diagnosis of SCI and persistent bowel dysfunction. Eligible patients were 18 years or older, had clinically recognized NBD after SCI, were able to participate in rehabilitation assessment and treatment, and had a relatively stable bowel management program before enrollment. NBD was assessed using the NBD score, a validated symptom-based instrument for SCI-related bowel dysfunction (14, 15).

Patients were excluded if they had bowel obstruction, active inflammatory bowel disease, known colorectal malignancy, acute severe anorectal disease, major colorectal surgery that would preclude assessment, severe cognitive or psychiatric impairment preventing cooperation with biofeedback, contraindications to electroacupuncture or electrical stimulation, active infection at the needle site, or incomplete baseline assessment.

### Interventions

All participants continued a standardized bowel-management program throughout the treatment period. The program included education regarding scheduled bowel care, individualized advice on dietary fiber and fluid intake, appropriate toileting position, abdominal massage, and continuation of previously prescribed oral laxatives, suppositories, enemas, digital stimulation, or manual evacuation when clinically required. Participants were required to have a relatively stable bowel regimen before enrollment, and any initiation, discontinuation, or dose modification of bowel-related medication during the study was recorded. The analysis records documented a planned 18 supervised biofeedback sessions over approximately 6 weeks, usually three sessions per week.

Instrumented anorectal biofeedback was delivered using a Solar GI high-resolution anorectal manometry system equipped with a visual biofeedback module and a rectal-balloon catheter (Laborie Medical Technologies, Portsmouth, NH, USA). The pressure channels were calibrated before each session. Participants were initially positioned in the left lateral position with the hips and knees flexed; when medically and functionally feasible, balloon-expulsion training was subsequently performed while seated on a commode chair. Each session lasted approximately 40–45 min and followed a standardized sequence: review of the bowel diary and home practice (approximately 5 min); diaphragmatic breathing and abdominal propulsion training (approximately 10 min); 10 simulated defecatory maneuvers with real-time rectal and anal pressure feedback, each lasting approximately 5–10 s with 30–60 s of rest (approximately 15 min); and rectal-balloon sensory and simulated evacuation training using 50 mL of tepid water, with a maximum of three expulsion attempts when safe (approximately 10 min). Graded balloon inflation and deflation were used in participants with rectal hyposensitivity. Participants were instructed to practice diaphragmatic breathing and coordinated defecatory maneuvers for 10–15 min twice daily. For patients with markedly impaired voluntary anal control, training emphasized abdominal propulsion, awareness of residual pressure changes, rectal sensory training, and reduction of paradoxical anal activation rather than conventional sphincter strengthening. Biofeedback was delivered by registered rehabilitation therapists or specialist rehabilitation nurses with at least 3 years of relevant experience who had completed standardized training in device operation, catheter placement, verbal instructions, and adverse-event management; procedural completion was recorded using a session checklist.

Participants in the combined-treatment group additionally received 18 electroacupuncture sessions over the same approximately 6-week period, usually three sessions per week. With the participant prone, sterile disposable needles measuring 0.30 × 75 mm were inserted obliquely toward the posterior sacral foramina at bilateral Shangliao (BL31), Ciliao (BL32), Zhongliao (BL33), and Xialiao (BL34). Approximate insertion depth was 40–50 mm at BL31 and BL32 and 30–40 mm at BL33 and BL34, with adjustment for body habitus and sacral anatomy. When sensation was preserved, needles were manipulated gently until a local dull, distending, or radiating sensation was reported. In patients with impaired sacral sensation, placement and stimulation intensity were guided by tissue resistance, visible rhythmic muscle response, and physiological tolerance. Electrical stimulation was delivered using an SDZ-V electroacupuncture stimulator (Hwato, Suzhou Medical Appliance Factory Co., Ltd., Suzhou, China). Corresponding left and right needles at each sacral level were connected as four electrode pairs. A continuous waveform at 50 Hz with a nominal pulse width of 0.20 ms was used; current was increased gradually from approximately 1 mA to a maximum of 5 mA according to tolerance, targeting the highest comfortable intensity that produced mild rhythmic contraction without pain, excessive spasm, or autonomic symptoms. Each session lasted 30 min. On days when both treatments were provided, biofeedback was completed first, followed by a 20–30-min rest interval before electroacupuncture.

Before each electroacupuncture session, the sacral skin was examined and participants were asked about pain, bleeding, dizziness, spasms, sleep disturbance, and symptoms suggestive of autonomic dysreflexia. For patients with cervical or upper thoracic SCI or a history of autonomic instability, blood pressure and heart rate were checked before and after electroacupuncture and anorectal physiological testing. Electroacupuncture was stopped for persistent pain, marked muscle spasm, dizziness, sweating, facial flushing, clinically relevant blood-pressure elevation, skin injury, uncontrolled bleeding, or other medically concerning reactions. Biofeedback was suspended for significant anorectal bleeding, severe pain, acute hemorrhoidal or fissure symptoms, suspected fecal impaction, or autonomic symptoms. Attendance, treatment parameters, completion of procedural components, participant tolerance, and adverse events were documented at each session. Treatment records were periodically reviewed by a senior rehabilitation clinician to assess protocol fidelity.

### Outcomes

The primary outcome was week 6 NBD score, analyzed with adjustment for the baseline NBD value. Lower scores indicate less severe bowel dysfunction. Key clinical secondary outcomes were spontaneous bowel movements per week, evacuation time, and PAC-QOL score. Evacuation time was recorded in the 7-day bowel diary as the patient-reported duration in minutes of each bowel-care episode; the mean across evaluable episodes was used. This measure reflected episode-level duration and did not quantify total weekly bowel-care burden or caregiver dependence. The PAC-QOL assessed constipation-related quality of life, with lower scores indicating better quality of life (16).

The key objective anorectal coordination outcome was anal relaxation during straining, expressed as a percentage. Anorectal physiological assessment was performed using the same manometry system and standardized verbal instructions at each visit and was scheduled at least 24 h after the most recent treatment session when feasible. Participants completed three simulated defecatory maneuvers, each lasting 15 s and separated by 30 s of rest. Anal relaxation was calculated as [(pre-strain anal pressure − residual anal pressure during strain) / pre-strain anal pressure] × 100, and the mean of three technically acceptable maneuvers was used. Positive values indicated anal pressure reduction during straining, whereas values near zero or below zero indicated absent relaxation or paradoxical contraction.

The exploratory physiological outcome was HRV RMSSD, expressed in milliseconds. HRV was recorded using a BIOPAC MP160 data-acquisition system with an ECG100C amplifier (BIOPAC Systems, Inc., Goleta, CA, USA) and a modified lead II configuration with three Ag/AgCl electrodes. Electrocardiographic signals were sampled at 1,000 Hz. Initial recordings lasted approximately 340–420 s, from which a 300-s segment of stable sinus rhythm was selected. Recordings were processed using Kubios HRV Scientific version 4.1.0 (Kubios Oy, Kuopio, Finland), retained as the fixed software version during the study. ECG traces and R-peak series were visually reviewed. Missed or additional detections and isolated ectopic beats were corrected using the medium 0.25-s threshold with cubic-spline interpolation. Segments were accepted when corrected beats comprised <=5% and no sustained arrhythmia or unresolved noise remained. Recordings were obtained at approximately the same time of day in a quiet, temperature-controlled room after at least 10 min of supine rest. Participants were instructed to avoid vigorous exercise, caffeine, nicotine, and a large meal before assessment. Respiratory pacing and the exact interval from the final electroacupuncture session were not available as standardized analysis variables for every recording; RMSSD was therefore interpreted as an exploratory correlate rather than evidence of a causal autonomic mechanism (26, 27).

Outcomes were assessed before treatment (week 0), immediately after the approximately 6-week treatment course (week 6), and approximately 6 weeks after treatment completion (week 12). Among participants completing the corresponding assessments, the median interval from baseline to week 6 was 45 days (IQR, 42–47; range, 40–49), and the median interval from baseline to week 12 was 90 days (IQR, 86–94; range, 82–99). Week 6 analyses used paired baseline and post-treatment values; week 12 analyses used paired baseline and follow-up values.

Participants and treating therapists were not blinded because the interventions were readily distinguishable. Whenever operationally feasible, outcome assessments were performed by personnel who were not directly responsible for treatment; however, a formal assessor-blinding procedure was not documented in the archived study records. Performance and detection bias are therefore possible, particularly for patient-reported outcomes.

### Statistical analysis

Continuous variables were summarized as mean ± standard deviation, and categorical variables as number and percentage. Baseline group comparisons used independent-samples t tests for continuous variables and chi-square or Fisher exact tests for categorical variables, as appropriate; these *p* values were descriptive because treatment was not randomized.

For week 6 analyses, multivariable ANCOVA was used for continuous outcomes. Each primary model included treatment group, the baseline value of the corresponding outcome, age, sex, neurological level, AIS grade, injury duration, and baseline bowel-management pattern. Adjusted differences were reported as EA + biofeedback minus biofeedback alone with 95% confidence intervals. Week 6 NBD score was the sole primary comparison. All other week 6 outcomes, week 12 analyses, longitudinal interactions, and additional *post-hoc* analyses were secondary or exploratory.

Propensity-score overlap weighting was used as a sensitivity analysis for non-randomized treatment selection. Propensity scores included age, sex, body mass index, smoking status, neurological level, AIS grade, injury duration, NBD duration, baseline bowel-management pattern, baseline NBD score, and baseline PAC-QOL score. Overlap-weighted outcome models included treatment group and the baseline outcome value with robust standard errors. Covariate balance, effective sample size, estimated propensity-score distributions, empirical common support, and overlap weights were examined (31, 32).

Covariate balance was evaluated using absolute standardized mean differences before and after weighting, with <0.10 indicating acceptable balance. Effective sample sizes, overlap-weight distributions, estimated propensity-score ranges, and empirical common support were also examined; diagnostics are reported in Supplementary Tables S4, S8 and Supplementary Figures S1, S2.

To assess model stability, the week 6 NBD estimate was compared across a baseline-adjusted model, a *post-hoc* parsimonious model, and the full primary model; stratified nonparametric bootstrap analyses with 2,000 repetitions were also performed for the full ANCOVA and overlap-weighted models. As a *post-hoc* multiplicity sensitivity analysis, Holm-adjusted *p* values were calculated separately for the five non-primary week 6 outcomes, 6 week 12 outcomes, and six longitudinal group-by-time interaction tests; the primary week 6 NBD comparison was not adjusted. NBD severity-category transitions were summarized descriptively. Balloon-expulsion time was analyzed as a *post-hoc* exploratory objective evacuation endpoint using baseline-adjusted raw-scale ANCOVA and a log-transformed sensitivity model. A *post-hoc* interaction analysis evaluated week 6 NBD effects by injury completeness. Exact supraconal, conal, and cauda-equina topography was not prospectively captured as a standardized analysis-ready variable for the full cohort and was not analyzed. Two-sided nominal *p* values and multiplicity-adjusted p values are reported according to this hierarchy.

Primary week 6 analyses used available paired observations because only three participants lacked week 6 outcome data. Week 12 analyses were supplemented by stabilized inverse-probability-of-follow-up weighting based on treatment group and baseline covariates. Reasons for unavailable assessments were summarized from follow-up records. Statistical analyses were performed using R software.

## Results

### Participant flow and baseline characteristics

**Figure 1:**
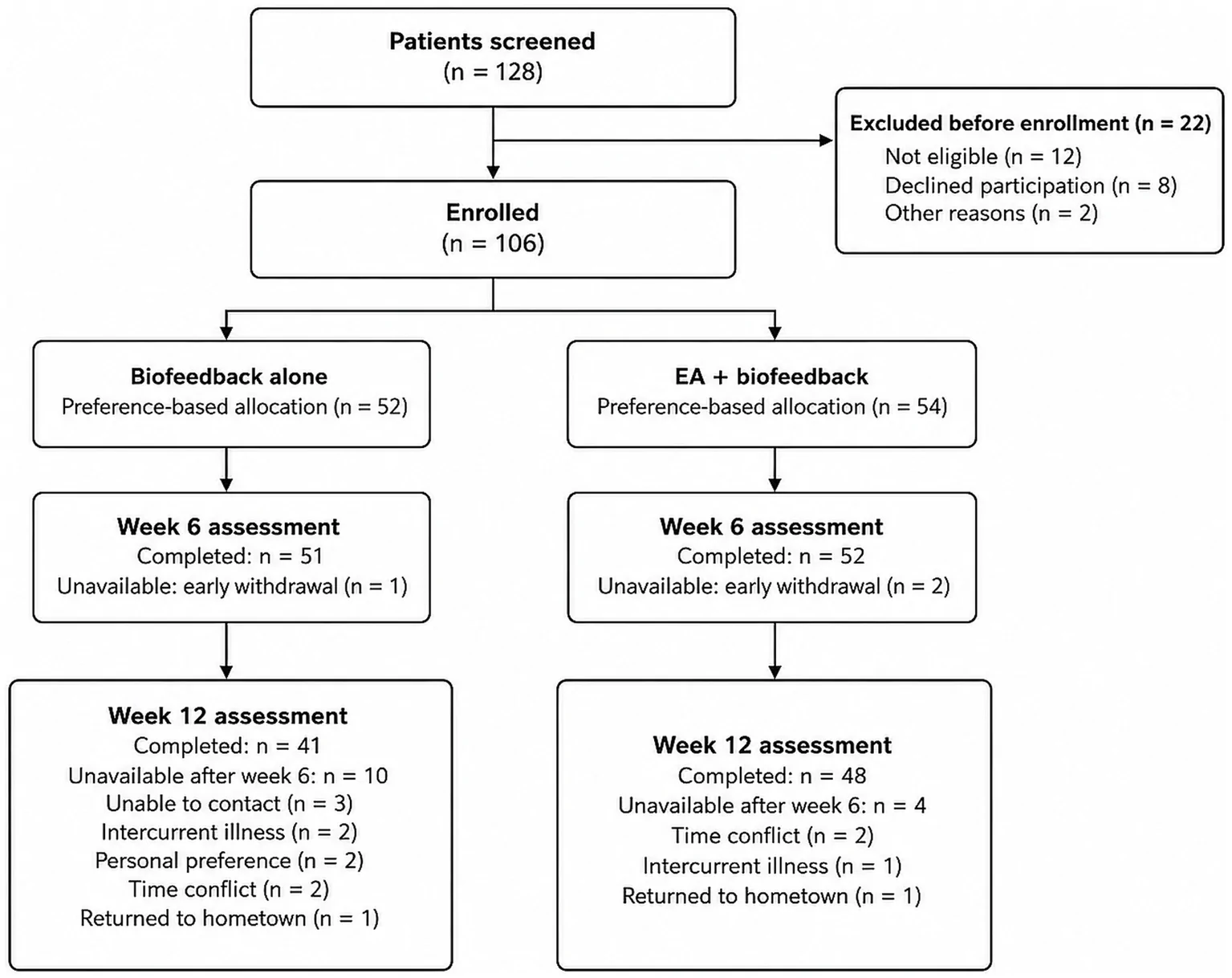
Study flowchart. The flowchart shows screening, reasons for exclusion before enrollment, preference-based treatment allocation, reasons for unavailable week 6 assessments, and reasons for unavailable week 12 assessments. EA, electroacupuncture.

A total of 128 patients were screened. Twenty-two were excluded before enrollment: 12 did not meet the inclusion criteria, 8 declined participation, and 2 were excluded for other reasons. Therefore, 106 patients were enrolled, including 52 in the biofeedback-alone group and 54 in the EA + biofeedback group. Week 6 assessment was completed by 51 and 52 patients, respectively; the unavailable assessments reflected early withdrawal (*n* = 1 and *n* = 2). Week 12 assessment was completed by 41 and 48 patients. Between week 6 and week 12, the biofeedback group had 10 unavailable assessments (unable to contact, *n* = 3; intercurrent illness, *n* = 2; personal preference, *n* = 2; time conflict, *n* = 2; returned to hometown, *n* = 1), and the combined-treatment group had 4 (time conflict, *n* = 2; intercurrent illness, *n* = 1; returned to hometown, *n* = 1). The median observed assessment times were 45 days from baseline to week 6 and 90 days from baseline to week 12 (Figure 1).

The overall mean age was 51.2 ± 12.1 years, and 81 patients (76%) were male. Traffic accident was the most common cause of SCI (44%), followed by fall injury (33%). Thoracic injury was the most frequent neurological level (52%), and 82% of patients had incomplete injury. Baseline NBD score was similar between groups (19.8 ± 3.6 in biofeedback alone vs. 20.4 ± 4.0 in EA + biofeedback; descriptive *p* = 0.4). Baseline PAC-QOL score was also comparable (2.2 ± 0.5 vs. 2.4 ± 0.5; descriptive *p* = 0.2). Baseline bowel management pattern differed between groups (descriptive *p* = 0.028), supporting the need for adjusted and sensitivity analyses (Table 1).

**Table 1:** Baseline characteristics of the full enrolled cohort.

Characteristic	Overall (*N* = 106)	Biofeedback alone (*n* = 52)	EA + biofeedback (*n* = 54)	*p* value
Age, years	51.2 ± 12.1	51.4 ± 12.1	51.1 ± 12.3	0.9
Sex				0.3
Female	25 (24%)	15 (29%)	10 (19%)	
Male	81 (76%)	37 (71%)	44 (81%)	
Body mass index, kg/m^2^	23.7 ± 3.0	24.0 ± 3.2	23.4 ± 2.7	0.3
Residence				0.3
Rural	47 (44%)	20 (38%)	27 (50%)	
Urban	59 (56%)	32 (62%)	27 (50%)	
Smoking status				0.088
Current/former	36 (34%)	13 (25%)	23 (43%)	
Never	70 (66%)	39 (75%)	31 (57%)	
Cause of spinal cord injury				0.9
Fall	35 (33%)	18 (35%)	17 (31%)	
Other	7 (6.6%)	4 (7.7%)	3 (5.6%)	
Sports/work injury	17 (16%)	9 (17%)	8 (15%)	
Traffic accident	47 (44%)	21 (40%)	26 (48%)	
Neurological level of injury				>0.9
Cervical	24 (23%)	11 (21%)	13 (24%)	
Thoracic	55 (52%)	27 (52%)	28 (52%)	
Lumbar/sacral	27 (25%)	14 (27%)	13 (24%)	
AIS grade				>0.9
A	19 (18%)	9 (17%)	10 (19%)	
B	19 (18%)	10 (19%)	9 (17%)	
C	41 (39%)	19 (37%)	22 (41%)	
D	27 (25%)	14 (27%)	13 (24%)	
Injury completeness				>0.9
Complete	19 (18%)	9 (17%)	10 (19%)	
Incomplete	87 (82%)	43 (83%)	44 (81%)	
Duration of injury, months	20.8 ± 11.1	22.6 ± 11.9	19.0 ± 10.1	0.090
Duration of neurogenic bowel dysfunction, months	19.2 ± 11.2	21.1 ± 12.1	17.3 ± 10.0	0.082
Baseline bowel management pattern				0.028
Manual evacuation	15 (14%)	6 (12%)	9 (17%)	
Mixed methods	20 (19%)	4 (7.7%)	16 (30%)	
Oral laxatives	23 (22%)	12 (23%)	11 (20%)	
Scheduled bowel program	25 (24%)	16 (31%)	9 (17%)	
Suppository/enema	23 (22%)	14 (27%)	9 (17%)	
Baseline NBD score	20.1 ± 3.8	19.8 ± 3.6	20.4 ± 4.0	0.4
Baseline PAC-QOL score	2.3 ± 0.5	2.2 ± 0.5	2.4 ± 0.5	0.2

Values are mean ± SD or n (%). This table uses the full enrolled cohort (*N* = 106). Continuous variables were compared using independent-samples t tests and categorical variables using chi-square or Fisher exact tests, as appropriate. *p* values are descriptive because treatment was not randomized. AIS, American Spinal Injury Association Impairment Scale; EA, electroacupuncture; NBD, neurogenic bowel dysfunction; PAC-QOL, Patient Assessment of Constipation Quality of Life.

### Clinical bowel and quality-of-life outcomes at post-treatment

The primary outcome favored the combined intervention. NBD score decreased from 20.8 ± 3.6 to 15.5 ± 4.2 in the EA + biofeedback group, compared with a decrease from 19.8 ± 3.6 to 16.9 ± 4.1 in the biofeedback-alone group. The mean change was −5.2 ± 2.9 points with combined treatment and −2.9 ± 1.9 points with biofeedback alone. After adjustment for baseline outcome value and the primary clinical covariates, the between-group difference was −2.1 points (95% CI, −3.2 to −1.1; *p* < 0.001; Table 2).

**Table 2:** Key clinical bowel and quality-of-life outcomes at week 6.

Outcome	BF, n; baseline → week 6	BF change	EA + BF, n; baseline → week 6	EA + BF change	Adjusted difference (95% CI)	*p* value
NBD score, points	*n* = 51; 19.8 ± 3.6 → 16.9 ± 4.1	-2.9 ± 1.9	*n* = 52; 20.8 ± 3.6 → 15.5 ± 4.2	−5.2 ± 2.9	−2.1 (−3.2 to −1.1)	<0.001
Spontaneous bowel movements, per week	*n* = 51; 2.2 ± 0.7 → 2.8 ± 0.9	0.6 ± 0.7	*n* = 52; 2.3 ± 0.7 → 3.2 ± 0.8	0.9 ± 0.7	0.3 (0.0 to 0.6)	0.043
Evacuation time, min	*n* = 51; 49.5 ± 14.8 → 40.6 ± 18.4	−8.9 ± 9.4	*n* = 52; 48.4 ± 13.0 → 36.1 ± 17.7	−12.3 ± 12.2	−3.3 (−8.1 to 1.4)	0.163
PAC-QOL score, score	*n* = 51; 2.25 ± 0.47 → 2.05 ± 0.59	−0.20 ± 0.30	*n* = 52; 2.37 ± 0.51 → 1.93 ± 0.59	−0.44 ± 0.35	−0.24 (−0.38 to −0.09)	0.001

Values are mean ± SD among participants with paired baseline and week 6 data (BF, *n* = 51; EA + BF, *n* = 52). Cells headed “baseline → week 6” show the actual values at both time points. Change was calculated as week 6 minus baseline. Adjusted differences represent EA + BF minus BF and were estimated using ANCOVA adjusted for baseline outcome value, age, sex, injury level, AIS grade, injury duration, and baseline bowel management pattern. Table 1 uses the full cohort; the outcome-specific denominator accounts for the small difference in baseline NBD means. AIS, American Spinal Injury Association Impairment Scale; BF, biofeedback; EA, electroacupuncture; NBD, neurogenic bowel dysfunction; PAC-QOL, Patient Assessment of Constipation Quality of Life.

**Figure 2:**
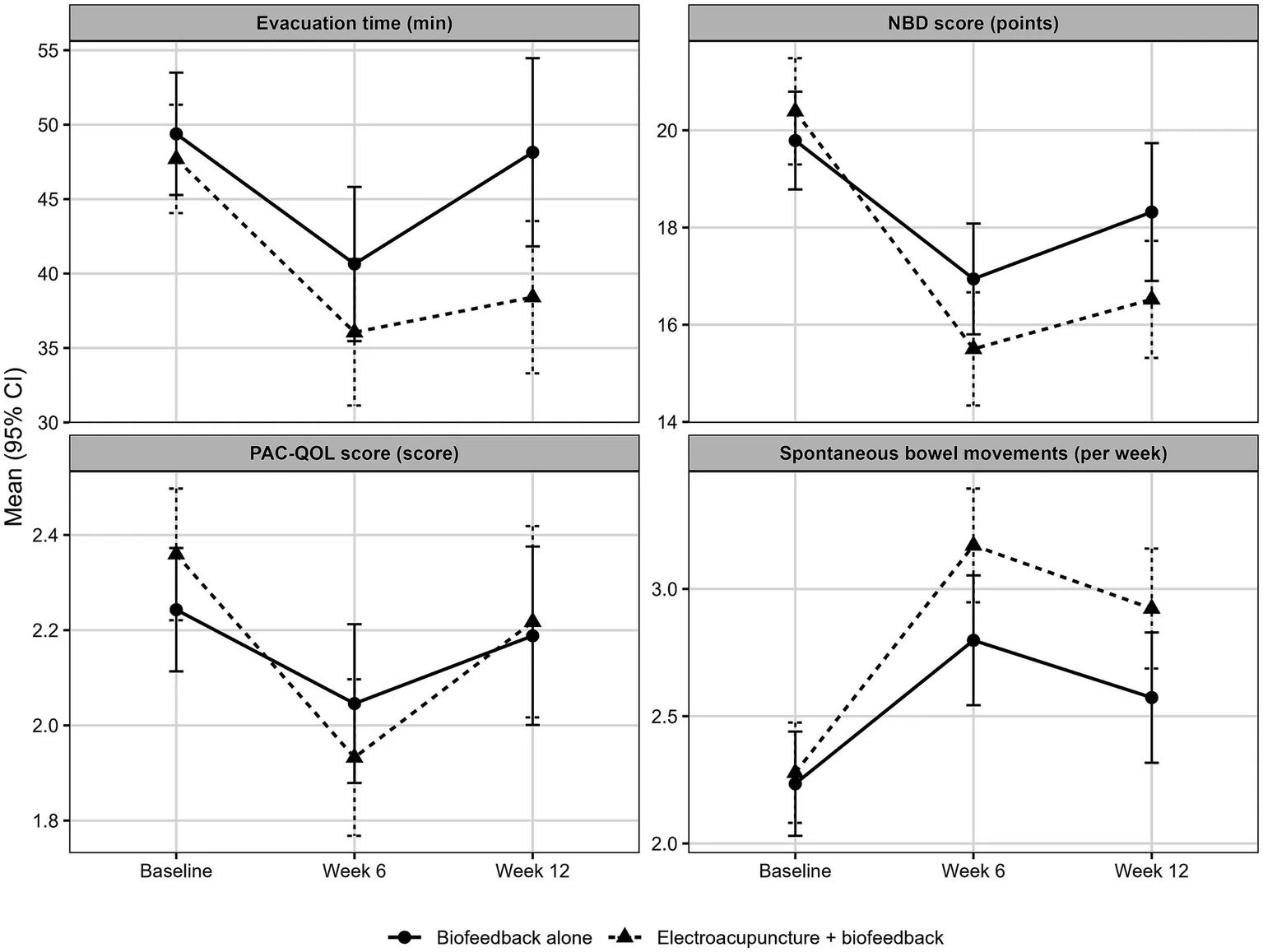
Longitudinal changes in key clinical bowel and quality-of-life outcomes at week 0, week 6, and week 12. Points represent group means and error bars represent 95% confidence intervals. Nominal *p* values are presented in the main tables; Holm-adjusted multiplicity results are reported in Supplementary Table S9. EA, electroacupuncture; NBD, neurogenic bowel dysfunction; PAC-QOL, Patient Assessment of Constipation Quality of Life.

Table 1 summarizes the full enrolled cohort, whereas Tables 2, 3 summarize participants with paired baseline and week 6 measurements. This outcome-specific denominator explains the small difference in the EA + biofeedback baseline NBD mean between Table 1 (*n* = 54) and Table 2 (*n* = 52; Figure 2).

**Table 3:** Key objective and exploratory physiological outcomes at week 6.

Outcome	BF, n; baseline → week 6	BF change	EA + BF, n; baseline → week 6	EA + BF change	Adjusted difference (95% CI)	*p* value
Anal relaxation during straining, %	*n* = 51; 11.1 ± 4.7 → 15.1 ± 7.7	4.0 ± 5.7	*n* = 52; 11.4 ± 5.5 → 20.1 ± 8.4	8.7 ± 6.6	4.9 (2.2 to 7.5)	<0.001
HRV RMSSD, ms	*n* = 51; 22.9 ± 5.2 → 25.2 ± 6.3	2.3 ± 3.5	*n* = 52; 22.5 ± 5.9 → 27.7 ± 6.9	5.2 ± 4.3	2.5 (0.8 to 4.2)	0.005

Values are mean ± SD among participants with paired baseline and week 6 data (BF, *n* = 51; EA + BF, *n* = 52). Cells headed “baseline → week 6” show the actual values at both time points. Change was calculated as week 6 minus baseline. Anal relaxation during straining was the focused objective anorectal coordination outcome. RMSSD was exploratory and does not establish a causal autonomic mechanism. Adjusted differences represent EA + BF minus BF and were estimated using ANCOVA adjusted for baseline outcome value and the primary clinical covariates. BF, biofeedback; EA, electroacupuncture; HRV, heart rate variability; RMSSD, root mean square of successive differences.

Spontaneous bowel movements increased in both groups, with a nominally larger increase in the combined-treatment group (adjusted difference, 0.3 per week; 95% CI, 0.0 to 0.6; nominal *p* = 0.043). This comparison did not remain below 0.05 after Holm adjustment across the five non-primary week 6 outcomes (adjusted *p* = 0.085). Evacuation time did not differ clearly at week 6 (adjusted difference, −3.3 min; 95% CI, −8.1 to 1.4; nominal *p* = 0.163).

PAC-QOL score improved more in the combined-treatment group (adjusted difference, −0.24; 95% CI, −0.38 to −0.09; nominal *p* = 0.001; Holm-adjusted *p* = 0.005). Anal relaxation during straining and RMSSD also remained below 0.05 after Holm adjustment at week 6 (adjusted *p* = 0.002 and 0.015, respectively), although RMSSD remained exploratory.

### Objective anorectal coordination and exploratory physiological outcomes

**Figure 3:**
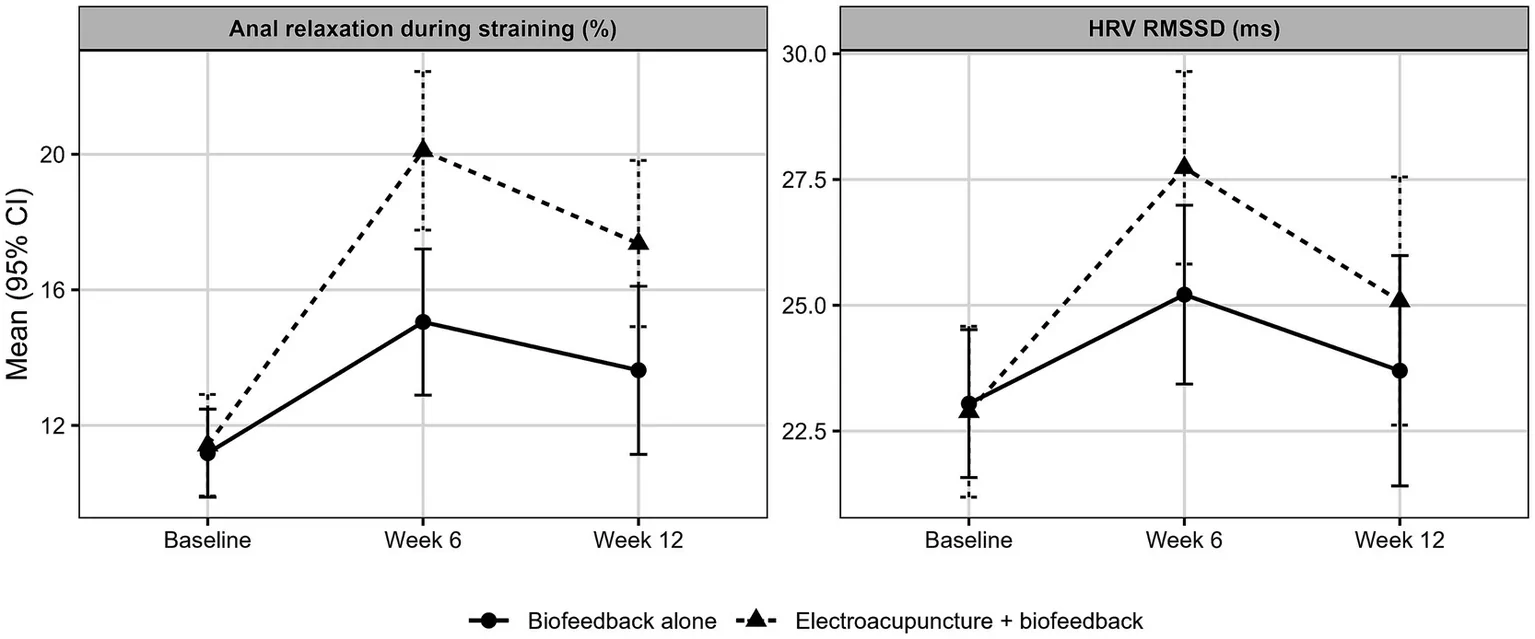
Longitudinal changes in anal relaxation during straining and HRV RMSSD at week 0, week 6, and week 12. Points represent group means and error bars represent 95% confidence intervals. Anal relaxation was the focused objective anorectal coordination outcome. RMSSD was exploratory and should not be interpreted as evidence of a causal autonomic mechanism. Holm-adjusted multiplicity results are reported in Supplementary Table S9. EA, electroacupuncture; HRV, heart rate variability; RMSSD, root mean square of successive differences.

Anal relaxation during straining improved in both groups, but the magnitude of improvement was larger in the combined-treatment group. The mean change was 8.7 ± 6.6% in the EA + biofeedback group and 4.0 ± 5.7% in the biofeedback-alone group. The adjusted between-group difference was 4.9% (95% CI, 2.2 to 7.5; *p* < 0.001), indicating greater improvement in the key objective anorectal coordination outcome (Table 3; Figure 3).

RMSSD increased from 22.5 ± 5.9 to 27.7 ± 6.9 ms in the combined-treatment group and from 22.9 ± 5.2 to 25.2 ± 6.3 ms in the biofeedback-alone group. The adjusted between-group difference was 2.5 ms (95% CI, 0.8 to 4.2; *p* = 0.005). We interpret this finding as an exploratory physiological correlate, not evidence of a causal autonomic mechanism (Table 3).

A total of 298 HRV assessments were retained for analysis: 106 at baseline, 103 at week 6, and 89 at week 12. Nine initial recordings required repeat acquisition because of inadequate signal quality. The median corrected-beat proportion in final 300-s segments was 0.26%, and all retained segments met the predefined quality-control criteria.

### Sensitivity, longitudinal, and follow-up analyses

The primary week 6 NBD estimate was similar across model specifications: −2.28 points (95% CI, −3.23 to −1.32) in the baseline-adjusted model, −2.06 (−3.07 to −1.04) in the parsimonious model, and −2.13 (−3.17 to −1.09) in the full model. All 2,000 bootstrap repetitions were successful; percentile 95% intervals were −3.22 to −1.02 for the full ANCOVA and −3.46 to −1.15 for the overlap-weighted model (Supplementary Table S7). Estimated propensity scores ranged from 0.067 to 0.904 in the biofeedback group and 0.089 to 0.958 in the combined-treatment group. Empirical common support was 0.089–0.904; 1/52 and 3/54 participants, respectively, lay outside this interval, indicating substantial but incomplete overlap (Supplementary Table S8; Supplementary Figure S2). After overlap weighting, all included covariates had absolute standardized mean differences <0.001, and the effective sample sizes were 41.0 and 41.3.

By week 6, 9/51 participants (17.6%) in the biofeedback group and 12/52 (23.1%) in the combined-treatment group improved by at least one NBD severity category; no participant worsened by category (Supplementary Table S10). In the *post-hoc* injury-completeness analysis, the adjusted NBD differences were −2.70 points in complete injury and −1.92 points in incomplete injury, with no evidence of interaction (P for interaction = 0.547); the complete-injury subgroup was small (Supplementary Table S12).

Balloon-expulsion time did not differ clearly at week 6 (adjusted difference, −10.2 s; 95% CI, −28.1 to 7.6; *p* = 0.258). At week 12, an exploratory raw-scale analysis yielded an adjusted difference of −18.6 s (95% CI, −34.8 to −2.5; nominal *p* = 0.025), with a consistent log-scale sensitivity estimate. Because this endpoint was post hoc and was not included in the original multiplicity family, it was interpreted as exploratory (Supplementary Table S11).

Mixed-effects models supported a differential longitudinal trajectory for NBD score (group-by-time *p* < 0.001), PAC-QOL (*p* = 0.011), anal relaxation during straining (*p* = 0.003), and RMSSD (*p* = 0.016). Spontaneous bowel movements showed a post-treatment difference but did not reach conventional statistical significance for the group-by-time interaction (*p* = 0.074). Evacuation time was not significantly different at post-treatment, but its longitudinal trajectory differed between groups (group-by-time *p* = 0.022; Table 4).

**Table 4:** Sensitivity and longitudinal analyses for selected outcomes.

Outcome	Primary adjusted estimate	Primary *p* value	PS overlap-weighted estimate	PS-weighted *p* value	Mixed-effects group-by-time *p* value
NBD score, points	−2.1 (−3.2 to −1.1)	<0.001	−2.3 (−3.3 to −1.2)	<0.001	<0.001
Spontaneous bowel movements, per week	0.3 (0.0 to 0.6)	0.043	0.4 (0.1 to 0.7)	0.011	0.074
Evacuation time, min	−3.3 (−8.1 to 1.4)	0.163	−2.8 (−8.0 to 2.3)	0.277	0.022
PAC-QOL score, score	−0.24 (−0.38 to −0.09)	0.001	−0.24 (−0.39 to −0.10)	0.001	0.011
Anal relaxation during straining, %	4.9 (2.2 to 7.5)	<0.001	5.3 (2.8 to 7.7)	<0.001	0.003
HRV RMSSD, ms	2.5 (0.8 to 4.2)	0.005	2.6 (0.9 to 4.3)	0.003	0.016

Estimates represent EA + BF minus BF. The propensity-score model included age, sex, body mass index, smoking status, injury level, AIS grade, injury duration, NBD duration, baseline bowel management pattern, baseline NBD score, and baseline PAC-QOL score. Overlap-weighted outcome models included treatment group and the baseline outcome value with robust standard errors. The maximum absolute standardized mean difference decreased from 0.58 before weighting to <0.001 after weighting; detailed balance is shown in Supplementary Table S4 and Supplementary Figure S1. Mixed-effects *p* values correspond to the group-by-time interaction. BF, biofeedback; EA, electroacupuncture; HRV, heart rate variability; NBD, neurogenic bowel dysfunction; PAC-QOL, Patient Assessment of Constipation Quality of Life; PS, propensity score; RMSSD, root mean square of successive differences.

At week 12, the NBD-score difference remained below the Holm-adjusted threshold (adjusted difference, −2.2 points; 95% CI, −3.5 to −0.9; nominal *p* = 0.001; Holm-adjusted *p* = 0.007). Nominal differences in evacuation time (−6.9 min; *p* = 0.010) and anal relaxation (3.5%; *p* = 0.038) did not remain below 0.05 after Holm adjustment (adjusted *p* = 0.052 and 0.153). Differences in spontaneous bowel movements, PAC-QOL, and RMSSD were also not supported after adjustment (Supplementary Tables S3, S9). IPCW estimates remained directionally similar, but attrition bias cannot be excluded.

### Treatment adherence and safety

Treatment adherence was high in both groups. Patients in the biofeedback-alone group completed 15.6 ± 2.1 biofeedback sessions, and those in the EA + biofeedback group completed 16.2 ± 1.3 biofeedback sessions and 16.2 ± 1.5 electroacupuncture sessions. Post-treatment visit completion was 98.1% in the biofeedback-alone group and 96.3% in the combined-treatment group; follow-up completion was 78.8 and 88.9%, respectively.

Adverse events were more frequent in the combined-treatment group. Mild local treatment-related discomfort occurred in 1/52 patients receiving biofeedback alone and 9/54 receiving combined treatment; minor bleeding or bruising occurred only in the combined-treatment group (4/54). Any adverse event occurred in 2/52 and 13/54 patients, respectively. No serious adverse events were observed (Supplementary Table S2).

## Discussion

Within this single-center preference-allocated cohort, the combined-treatment group showed a modestly larger week 6 reduction in NBD score than the biofeedback-alone group. The estimate was similar across baseline-adjusted, parsimonious, full, overlap-weighted, and bootstrap analyses. However, these analyses address only measured covariates and cannot isolate an electroacupuncture-specific contribution or exclude unmeasured confounding related to treatment preference, expectations, perceived burden, cost, or access. The NBD score captures clinically important bowel symptoms, but its psychometric limitations and the absence of an established minimal clinically important difference require cautious interpretation (14, 15).

The study should therefore be interpreted as a pragmatic feasibility-and-association signal rather than evidence of an established additive effect. The combined-treatment arm received the full biofeedback program plus additional clinician-delivered electroacupuncture sessions, resulting in greater treatment time, therapist contact, attention, and expectation. Without sham electroacupuncture or an attention-matched control, nonspecific treatment effects cannot be separated from any specific effect of sacral-region stimulation.

A definitive minimal clinically important difference has not been established for the NBD score, and one validation study reported limited internal consistency (14, 15). The adjusted 2.1-point difference should therefore be interpreted cautiously. At week 6, mean scores remained within the severe range in both groups. Only 17.6% of the biofeedback group and 23.1% of the combined-treatment group improved by at least one severity category, and no participant worsened by category. These findings indicate relative symptom improvement rather than normalization or comprehensive remission.

The improvement in PAC-QOL supports a parallel change in constipation-related quality-of-life score. NBD is not only a physiological problem but also a daily-life problem: long bowel-care sessions, fear of accidents, and reliance on assistance can shape patient autonomy and social participation (4, 8). The PAC-QOL was originally designed to quantify the burden of constipation on everyday functioning and well-being (16). In the present study, its post-treatment improvement occurred in the same direction as the reduction in NBD score, but the absence of an established minimal clinically important difference in this population precludes claiming that the adjusted mean difference was individually perceptible or clinically decisive.

Spontaneous bowel movements showed a small nominal between-group difference at week 6, but this comparison did not remain below 0.05 after Holm adjustment. Accordingly, bowel frequency should be interpreted as directionally supportive rather than a statistically robust secondary benefit. The magnitude, approximately 0.3 bowel movements per week, is unlikely to be clinically decisive on its own.

Evacuation time showed no clear week 6 difference. Nominal week 12 and longitudinal results suggested a possible later separation, but the week 12 comparison did not remain below 0.05 after Holm adjustment. Likewise, the *post-hoc* balloon-expulsion analysis was negative at week 6 and nominally favorable at week 12. These objective emptying findings are exploratory and do not establish a durable treatment effect.

Anal relaxation during straining provides an objective physiological correlate, but it may still depend on participant effort and assessor knowledge. Biofeedback is used to retrain rectoanal coordination in functional defecatory disorders, and selected patients with incomplete SCI may retain sufficient voluntary or reflexive control for coordination training (17–19). Its week 6 difference remained below the Holm-adjusted threshold, whereas the week 12 difference did not. The finding supports further study of rectoanal coordination but does not establish that the combined intervention caused the physiological change.

The choice to focus on anal relaxation rather than a broad panel of anorectal manometry variables was deliberate. Anorectal physiology testing can identify abnormalities in tone, contractility, rectoanal coordination, and rectal sensation, but interpretation becomes difficult when multiple correlated parameters are tested without a clear hypothesis (25). Recent reviews of neurogenic bowel research have also noted that objective measures are heterogeneous and not uniformly integrated into treatment studies (13). By retaining a single coordination-oriented measure, this study provides objective support while avoiding an unfocused physiological narrative.

The RMSSD finding remains exploratory despite the more detailed acquisition and quality-control procedures. High-frequency ECG acquisition, standardized segment selection, visual review, and low artifact burden improve technical reproducibility, but RMSSD remains sensitive to respiration, medications, sleep, emotional state, physical activity, and timing relative to treatment (26–28, 33). The week 6 difference remained below the Holm-adjusted threshold, but it was not maintained at week 12 and cannot establish autonomic mediation.

The possible neuromodulatory role of Baliao electroacupuncture is biologically plausible but remains incompletely established. Baliao points are located over the sacral region, anatomically close to neural structures involved in pelvic organ function. Acupuncture and electroacupuncture have been studied in SCI rehabilitation and pelvic organ dysfunction, but systematic reviews emphasize that evidence quality varies and sham-controlled studies remain limited (21). A prospective observational study reported improvements in bladder and bowel function after electroacupuncture in traumatic SCI (22). However, the present study cannot separate point-specific effects, nonspecific treatment effects, therapist contact, or patient expectation from true sacral neuromodulation.

Combining electroacupuncture with biofeedback is a conceptually plausible strategy for further study because the interventions target different components of bowel rehabilitation (18, 22). Nevertheless, the current comparison does not distinguish modality-specific effects from added therapist contact, attention, procedural burden, and expectation. The results therefore do not demonstrate causal superiority of the combined pathway.

No serious adverse events were observed, but the combined-treatment group had more minor adverse events, principally local treatment-related discomfort and bruising. The sample was too small to establish safety for uncommon events. In clinical practice, bowel and pelvic procedures in patients with SCI still require monitoring for intolerance and autonomic dysreflexia, particularly in higher-level injuries.

Several important limitations remain. Treatment allocation was preference-based rather than randomized, baseline bowel-management patterns differed, and overlap weighting cannot address unmeasured confounding. The full primary model also had a limited observations-to-parameter ratio; model-specification and bootstrap analyses reduced but did not eliminate concerns about instability. The study was not prospectively registered and had no formal *a priori* sample-size calculation. Participants and therapists were unblinded, formal assessor blinding could not be verified, and patient-reported outcomes were vulnerable to expectation and detection bias. Follow-up extended only approximately 6 weeks after treatment completion, attrition was differential, and several nominal week 12 differences did not withstand multiplicity adjustment. HRV also remains sensitive to unmeasured respiratory and contextual factors (26, 27).

Additional measurement limitations should be acknowledged. Exact supraconal, conal, and cauda-equina topography was not prospectively captured using a standardized analysis-ready variable for the full cohort, so only the reliably recorded complete-versus-incomplete classification was used for a *post-hoc* interaction analysis. Total weekly bowel-care time and caregiver dependence were not prospectively measured as standardized longitudinal outcomes and could not be reconstructed reliably for formal analysis. Risk-based monitoring for autonomic dysreflexia was performed, but event-level surveillance data were not captured in a standardized research dataset that permitted reliable incidence or treatment-attribution analyses. No serious adverse event was recorded in the study-wide safety dataset, but an autonomic-dysreflexia-specific incidence estimate could not be derived reliably. The detailed anorectal catheter configuration, respiratory pacing, and exact interval between every HRV recording and the last electroacupuncture session could not be verified from retained records.

External validity is limited by the single-center Chinese setting, the predominance of men, and the high proportion of incomplete and thoracic injuries. The complete-injury subgroup was small, and the absence of a statistically detectable interaction should not be interpreted as equivalence across lesion phenotypes. A recent prospective study in motor-incomplete SCI reported no improvement in anorectal manometric parameters after a biofeedback program, further underscoring heterogeneity across protocols and patient populations (34). Effects may differ in women, patients with complete injury, precisely characterized conal or cauda-equina lesions, and healthcare systems with different bowel-rehabilitation pathways.

The study nevertheless addresses an under-studied rehabilitation problem, combines patient-reported outcomes with focused anorectal and exploratory physiological measures, and provides transparent sensitivity analyses using established symptom and quality-of-life instruments (14, 16). Its main contribution is a pragmatic signal that can inform the design of a future randomized, prospectively registered trial with an electroacupuncture-only arm, sham or attention-matched control, standardized lesion-topography classification, prespecified patient-burden outcomes, systematic autonomic-dysreflexia surveillance, blinded assessment, and longer follow-up.

In conclusion, the combined-treatment pathway was associated with a modestly larger week 6 reduction in NBD score, and the primary estimate was stable across several analytic specifications. Secondary and objective findings were mixed after multiplicity adjustment, mean NBD scores remained in the severe range, and only the NBD difference remained below the adjusted threshold at week 12. Given preference-based allocation, additional treatment contact, possible unmeasured confounding, absent prospective registration, limited sample-size planning, unverified assessor blinding, incomplete standardized measurement of several clinically important domains, and short follow-up, the findings are hypothesis-generating rather than evidence of an electroacupuncture-specific additive effect, causal superiority, or durable benefit.

## Conclusion

In this single-center preference-allocated study, adding Baliao electroacupuncture to anorectal biofeedback was associated with a modestly larger short-term reduction in NBD score. The findings do not isolate an electroacupuncture-specific contribution or establish causal superiority or durable benefit; secondary, subgroup, balloon-expulsion, and HRV results should be regarded as exploratory.

## Data Availability

The raw data supporting the conclusions of this article will be made available by the authors, without undue reservation.
