# Correction: Electroacupuncture at Baliao points added to anorectal biofeedback for spinal cord injury–related neurogenic bowel dysfunction: a single-center prospective preference-allocated comparative study

**DOI:** 10.3389/fmed.2026.2008883

**Published:** 2026-10-13

**Authors:** Mingxuan Liao, Xiao Huang, Dan Mo

**Affiliations:** General Hospital of the Yangtze River Shipping, Wuhan Brain Hospital, Wuhan, China

**Keywords:** anorectal biofeedback, Baliao points, electroacupuncture, heart rate variability, neurogenic bowel dysfunction, rehabilitation, spinal cord injury

The funder Traditional Chinese Medicine Research Project of Wuhan Municipal Health Commission, Grant No. WZ22Q02 to Mingxuan Liao was erroneously omitted.

The original version of this article has been updated.

