# Supplementary material for: Electroacupuncture at Baliao points added to anorectal biofeedback for spinal cord injury–related neurogenic bowel dysfunction: a single-center prospective preference-allocated comparative study
Source: Front Med (Lausanne). 2026 Aug 12;13:1866886. doi: 10.3389/fmed.2026.1866886 (PMC13506433; doi:10.3389/fmed.2026.1866886)
Supplement: Supplementary file 1 [file Table_1.docx]

**Supplementary Materials**

# Supplementary Table S1. Treatment adherence and assessment completion

| **Group** | **Biofeedback sessions completed, mean ± SD** | **Electroacupuncture sessions completed, mean ± SD** | **Week 6 completed, n (%)** | **Week 12 completed, n (%)** |
| --- | --- | --- | --- | --- |
| Biofeedback alone | 15.6 ± 2.1 | 0.0 ± 0.0 | 51/52 (98.1%) | 41/52 (78.8%) |
| EA + biofeedback | 16.2 ± 1.3 | 16.2 ± 1.5 | 52/54 (96.3%) | 48/54 (88.9%) |

Note: The protocol planned 18 sessions over approximately 6 weeks. The value 0.0 ± 0.0 for electroacupuncture in the biofeedback-alone group reflects the intervention design rather than a measured treatment exposure. EA, electroacupuncture.

# Supplementary Table S2. Adverse events

| **Adverse event** | **Biofeedback alone (n = 52)** | **EA + biofeedback (n = 54)** |
| --- | --- | --- |
| Mild local treatment-related discomfort | 1 (1.9%) | 9 (16.7%) |
| Minor bleeding or bruise | 0 (0.0%) | 4 (7.4%) |
| Dizziness | 0 (0.0%) | 0 (0.0%) |
| Transient bowel symptom worsening | 1 (1.9%) | 3 (5.6%) |
| Serious adverse event | 0 (0.0%) | 0 (0.0%) |
| Any adverse event | 2 (3.8%) | 13 (24.1%) |

Note: Events are patient-level counts. Mild local treatment-related discomfort included transient discomfort associated with either rehabilitation procedure. EA, electroacupuncture.

# Supplementary Table S3. Core outcomes at week 12

| **Outcome** | **BF baseline → week 12** | **BF change** | **EA + BF baseline → week 12** | **EA + BF change** | **Adjusted difference (95% CI)** | **P value** |
| --- | --- | --- | --- | --- | --- | --- |
| NBD score, points | 20.1 ± 3.9 → 18.3 ± 4.5 | -1.8 ± 2.4 | 20.8 ± 3.6 → 16.5 ± 4.1 | -4.3 ± 3.1 | -2.2 (-3.5 to -0.9) | 0.001 |
| Spontaneous bowel movements, per week | 2.2 ± 0.7 → 2.6 ± 0.8 | 0.4 ± 0.6 | 2.3 ± 0.7 → 2.9 ± 0.8 | 0.6 ± 0.7 | 0.3 (-0.0 to 0.6) | 0.095 |
| Evacuation time, min | 50.4 ± 15.2 → 48.1 ± 20.0 | -2.3 ± 11.8 | 48.0 ± 13.2 → 38.4 ± 17.6 | -9.6 ± 13.1 | -6.9 (-12.1 to -1.7) | 0.01 |
| PAC-QOL score, score | 2.27 ± 0.48 → 2.19 ± 0.59 | -0.08 ± 0.40 | 2.39 ± 0.50 → 2.22 ± 0.69 | -0.17 ± 0.38 | -0.04 (-0.22 to 0.14) | 0.641 |
| Anal relaxation during straining, % | 10.9 ± 4.9 → 13.6 ± 7.9 | 2.7 ± 7.4 | 11.5 ± 5.7 → 17.4 ± 8.5 | 5.9 ± 6.7 | 3.5 (0.2 to 6.9) | 0.038 |
| HRV RMSSD, ms | 23.0 ± 5.5 → 23.7 ± 7.3 | 0.7 ± 5.1 | 22.9 ± 6.0 → 25.1 ± 8.5 | 2.2 ± 5.1 | 1.0 (-1.4 to 3.4) | 0.392 |

Note: Values are mean ± SD among participants with paired baseline and week 12 data (BF, n = 41; EA + BF, n = 48). Adjusted differences represent EA + BF minus BF and were estimated using ANCOVA adjusted for baseline outcome value, age, sex, injury level, AIS grade, injury duration, and baseline bowel management pattern. BF, biofeedback; EA, electroacupuncture; HRV, heart rate variability; NBD, neurogenic bowel dysfunction; PAC-QOL, Patient Assessment of Constipation Quality of Life; RMSSD, root mean square of successive differences.

# Supplementary Table S4. Covariate balance before and after propensity-score overlap weighting

| **Covariate or level** | **Unweighted SMD** | **Overlap-weighted SMD** |
| --- | --- | --- |
| Age, years | -0.027 | -0.000 |
| Body mass index, kg/m² | -0.224 | -0.000 |
| Injury duration, months | -0.333 | -0.000 |
| NBD duration, months | -0.342 | -0.000 |
| Baseline NBD score | 0.157 | 0.000 |
| Baseline PAC-QOL score | 0.239 | 0.000 |
| Sex: Male | 0.242 | 0.000 |
| Smoking status: Never | -0.375 | -0.000 |
| Neurological level: Cervical | 0.069 | -0.000 |
| Neurological level: Lumbar/sacral | -0.065 | -0.000 |
| Neurological level: Thoracic | -0.001 | 0.000 |
| AIS grade: A | 0.031 | 0.000 |
| AIS grade: B | -0.066 | -0.000 |
| AIS grade: C | 0.086 | -0.000 |
| AIS grade: D | -0.065 | 0.000 |
| Baseline bowel management: Manual evacuation | 0.146 | 0.000 |
| Baseline bowel management: Mixed methods | 0.581 | 0.000 |
| Baseline bowel management: Oral laxatives | -0.065 | -0.000 |
| Baseline bowel management: Scheduled bowel program | -0.333 | -0.000 |
| Baseline bowel management: Suppository/enema | -0.248 | -0.000 |

Note: SMDs are expressed as EA + biofeedback minus biofeedback alone. Absolute SMD <0.10 was used to define acceptable balance. The maximum absolute SMD decreased from 0.581 before weighting to <0.001 after overlap weighting. Effective sample sizes were 41.0 for biofeedback alone and 41.3 for EA + biofeedback. AIS, American Spinal Injury Association Impairment Scale; NBD, neurogenic bowel dysfunction; PAC-QOL, Patient Assessment of Constipation Quality of Life; SMD, standardized mean difference.

# Supplementary Table S5. Baseline balance between week 12 completers and noncompleters

| **Covariate or level** | **SMD: completers vs. noncompleters** |
| --- | --- |
| Age, years | -0.019 |
| Body mass index, kg/m² | 0.155 |
| Injury duration, months | -0.051 |
| NBD duration, months | -0.051 |
| Baseline NBD score | 0.636 |
| Baseline PAC-QOL score | 0.420 |
| Sex: Male | -0.002 |
| Smoking status: Never | 0.175 |
| Neurological level: Cervical | 0.335 |
| Neurological level: Lumbar/sacral | 0.053 |
| Neurological level: Thoracic | -0.307 |
| AIS grade: A | 0.201 |
| AIS grade: B | 0.201 |
| AIS grade: C | -0.060 |
| AIS grade: D | -0.254 |
| Baseline bowel management: Manual evacuation | -0.113 |
| Baseline bowel management: Mixed methods | 0.757 |
| Baseline bowel management: Oral laxatives | -0.363 |
| Baseline bowel management: Scheduled bowel program | 0.002 |
| Baseline bowel management: Suppository/enema | -0.051 |

Note: Positive SMDs indicate higher values or proportions among week 12 completers. The maximum absolute SMD was 0.757, indicating that complete-case follow-up estimates may be vulnerable to attrition bias. SMD, standardized mean difference.

# Supplementary Table S6. Inverse-probability-of-follow-up-weighted sensitivity analysis at week 12

| **Outcome** | **IPCW-adjusted difference (95% CI)** | **P value** |
| --- | --- | --- |
| NBD score, points | -2.1 (-3.2 to -0.9) | <0.001 |
| Spontaneous bowel movements, per week | 0.2 (-0.1 to 0.5) | 0.133 |
| Evacuation time, min | -6.9 (-11.5 to -2.3) | 0.003 |
| PAC-QOL score, score | -0.05 (-0.22 to 0.11) | 0.521 |
| Anal relaxation during straining, % | 3.5 (0.6 to 6.4) | 0.019 |
| HRV RMSSD, ms | 0.9 (-1.2 to 2.9) | 0.422 |

Note: Stabilized inverse-probability-of-follow-up weights were estimated from treatment group, age, sex, body mass index, smoking status, injury level, AIS grade, injury duration, NBD duration, baseline bowel management, baseline NBD score, and baseline PAC-QOL score. Weighted models additionally adjusted for the baseline outcome and primary clinical covariates. IPCW, inverse probability of censoring/follow-up weighting.

**Supplementary Table S7. Model-specification and bootstrap stability analyses for the week 6 NBD outcome**

| **Model** | **N** | **Slope df** | **Observations/slope df** | **Adjusted difference (95% CI)** | **P value** |
| --- | --- | --- | --- | --- | --- |
| Baseline-adjusted | 103 | 2 | 51.5 | -2.28 (-3.23 to -1.32) | <0.001 |
| Parsimonious sensitivity | 103 | 8 | 12.9 | -2.06 (-3.07 to -1.04) | <0.001 |
| Full primary model | 103 | 14 | 7.4 | -2.13 (-3.17 to -1.09) | <0.001 |

| **Analysis** | **Successful/attempted** | **Bootstrap mean** | **Percentile 95% CI** |
| --- | --- | --- | --- |
| Full ANCOVA | 2000/2000 | -2.13 | -3.22 to -1.02 |
| Full overlap-weighted model | 2000/2000 | -2.29 | -3.46 to -1.15 |

Note: The baseline-adjusted model included treatment group and baseline NBD score. The parsimonious model included treatment group, baseline NBD score, injury completeness, injury duration, and baseline bowel-management pattern. The full model used the primary covariate set reported in the main analysis. Bootstrap analyses used 2,000 treatment-stratified repetitions. These were post hoc sensitivity analyses.

**Supplementary Table S8. Propensity-score distribution and empirical common support**

| **Group** | **N** | **Propensity-score range** | **Median (IQR)** | **Overlap-weight range** | **Effective sample size** | **Outside common support, n (%)** |
| --- | --- | --- | --- | --- | --- | --- |
| Biofeedback alone | 52 | 0.067-0.904 | 0.387 (0.223-0.511) | 0.067-0.904 | 41.0 | 1 (1.9%) |
| EA + biofeedback | 54 | 0.089-0.958 | 0.640 (0.487-0.790) | 0.042-0.911 | 41.3 | 3 (5.6%) |

Note: The empirical common-support interval was 0.089-0.904. These results indicate substantial but incomplete propensity-score overlap. Covariate balance is shown in Supplementary Table S4 and Supplementary Figure S1; the full score distributions are shown in Supplementary Figure S2.

**Supplementary Table S9. Post hoc multiplicity sensitivity analysis**

| **Outcome** | **Hierarchy** | **Week 6 nominal P** | **Week 6 Holm P** | **Week 12 nominal P** | **Week 12 Holm P** | **Longitudinal nominal P** | **Longitudinal Holm P** |
| --- | --- | --- | --- | --- | --- | --- | --- |
| NBD score | Primary | <0.001 | Not adjusted | 0.001 | 0.007 | <0.001 | <0.001 |
| Spontaneous bowel movements | Secondary | 0.043 | 0.085 | 0.095 | 0.284 | 0.074 | 0.074 |
| Evacuation time | Secondary | 0.163 | 0.163 | 0.010 | 0.052 | 0.022 | 0.047 |
| PAC-QOL score | Secondary | 0.001 | 0.005 | 0.641 | 0.784 | 0.011 | 0.046 |
| Anal relaxation during straining | Secondary | <0.001 | 0.002 | 0.038 | 0.153 | 0.003 | 0.016 |
| HRV RMSSD | Exploratory | 0.005 | 0.015 | 0.392 | 0.784 | 0.016 | 0.047 |

Note: Week 6 NBD score was the sole primary comparison and was not multiplicity adjusted. Holm adjustment was applied separately to the five non-primary week 6 outcomes, the six week 12 outcomes, and the six longitudinal interaction tests. This analysis was post hoc and is intended to assess robustness rather than redefine the original analysis hierarchy.

**Supplementary Table S10. NBD severity-category transitions from baseline to week 6**

| **Group** | **Baseline category** | **Very minor (0-6)** | **Minor (7-9)** | **Moderate (10-13)** | **Severe (>=14)** |
| --- | --- | --- | --- | --- | --- |
| Biofeedback alone | Moderate (10-13) | 1 (50.0%) | 0 (0.0%) | 1 (50.0%) | 0 (0.0%) |
| Biofeedback alone | Severe (>=14) | 0 (0.0%) | 1 (2.0%) | 7 (14.3%) | 41 (83.7%) |
| EA + biofeedback | Moderate (10-13) | 0 (0.0%) | 0 (0.0%) | 1 (100.0%) | 0 (0.0%) |
| EA + biofeedback | Severe (>=14) | 2 (3.9%) | 3 (5.9%) | 7 (13.7%) | 39 (76.5%) |

| **Group** | **Improved by >=1 category** | **Unchanged** | **Worsened** |
| --- | --- | --- | --- |
| Biofeedback alone | 9/51 (17.6%) | 42/51 (82.4%) | 0/51 (0.0%) |
| EA + biofeedback | 12/52 (23.1%) | 40/52 (76.9%) | 0/52 (0.0%) |

Note: NBD severity categories were very minor (0-6), minor (7-9), moderate (10-13), and severe (>=14). This post hoc descriptive analysis contextualizes the clinical magnitude of the primary NBD-score difference.

**Supplementary Table S11. Post hoc exploratory balloon-expulsion-time analysis**

| **Time point** | **BF, n; baseline -> endpoint** | **EA+BF, n; baseline -> endpoint** | **Adjusted mean difference (95% CI), s** | **Nominal P** | **Geometric-mean ratio (95% CI)** | **Log-scale P** |
| --- | --- | --- | --- | --- | --- | --- |
| Week 6 | n=51; 177.2 ± 46.2 -> 158.0 ± 53.8 | n=52; 190.4 ± 53.6 -> 160.6 ± 67.2 | -10.2 (-28.1 to 7.6) | 0.258 | 0.88 (0.76 to 1.01) | 0.063 |
| Week 12 | n=41; 176.8 ± 46.5 -> 165.1 ± 55.6 | n=48; 188.5 ± 54.5 -> 160.6 ± 67.0 | -18.6 (-34.8 to -2.5) | 0.025 | 0.82 (0.71 to 0.94) | 0.007 |

Note: Negative raw-scale differences and geometric-mean ratios below 1 favor EA + biofeedback. Models adjusted for the baseline balloon-expulsion value and the primary clinical covariates. This endpoint was post hoc and was not included in the original multiplicity family; results should therefore be interpreted cautiously.

**Supplementary Table S12. Post hoc exploratory interaction analysis of week 6 NBD score by injury completeness**

| **Subgroup category** | **Subgroup level** | **BF, n** | **EA+BF, n** | **Adjusted difference (95% CI)** | **P value** | **P for interaction** |
| --- | --- | --- | --- | --- | --- | --- |
| Injury completeness | Complete | 9 | 10 | -2.70 (-5.04 to -0.35) | 0.025 | 0.547 |
|  | Incomplete | 42 | 42 | -1.92 (-3.04 to -0.79) | 0.001 |  |

Note: The interaction P value was obtained from a model containing treatment group, injury completeness, and the treatment-by-completeness interaction, with baseline NBD score and the parsimonious covariate set. The complete-injury subgroup was small; subgroup estimates are exploratory and should not be interpreted as evidence of equivalence. Exact supraconal/conal/cauda-equina topography was not analyzed because it was not prospectively captured as a standardized analysis-ready variable for the full cohort.

**Supplementary Figure S1. Covariate balance before and after propensity-score overlap weighting**

Supplementary Figure S1. Love plot of absolute standardized mean differences before and after overlap weighting. The vertical dashed line marks the balance threshold of 0.10. All included covariates were below this threshold after weighting.

**Supplementary Figure S2. Distribution of estimated propensity scores before and after overlap weighting**

Note: Panel A shows the unweighted propensity-score distributions and empirical common-support boundaries. Panel B shows the weighted distributions after overlap weighting. The distributions demonstrate substantial but incomplete initial overlap and close alignment after weighting.
